# Sequential Early-Life Infections Alter Peripheral Blood Transcriptomics in Aging Female Mice but Not the Response to De Novo Infection with Influenza Virus or *M. tuberculosis*

**DOI:** 10.4049/immunohorizons.2200066

**Published:** 2023-08-09

**Authors:** Kathleen G. Lanzer, Tres Cookenham, Elin Lehrmann, Yongqing Zhang, Debbie Duso, Qingqing Xie, William W. Reiley, Kevin G. Becker, Marcia A. Blackman

**Affiliations:** * Trudeau Institute, Saranac Lake, NY; † Computational Biology and Genomics Core, Laboratory of Genetics and Genomics, National Institute on Aging Intramural Research Program, National Institutes of Health, Baltimore, MD

## Abstract

To determine the impact of accumulating Ag exposure on immunity in the aging mouse, and to develop a model more relevant to humans who are exposed to multiple pathogens during life, we sequentially infected young female mice with four distinct pathogens at 8-wk intervals: murine γ-herpesvirus 68, Sendai virus, murine CMV, and *Heligmosomoides polygyrus*. Mock-infected mice received PBS. After aging the sequentially infected and mock-infected mice to 18–25 mo under specific pathogen-free conditions, we analyzed multiple immune parameters. We assessed transcriptional activity in peripheral blood, T cell phenotype, the diversity of influenza epitopes recognized by CD8 T cells, and the response of the animals to infection with influenza virus and *Mycobacterium tuberculosis*. Our data show enhanced transcriptional activation in sequentially infected aged mice, with changes in some CD8 T cell subsets. However, there was no measurable difference in the response of mock-infected and sequentially infected aged mice to de novo infection with either influenza virus or *M. tuberculosis* at 18–21 mo. Unexpectedly, a single experiment in which 25-mo-old female mice were challenged with influenza virus revealed a significantly higher survival rate for sequentially infected (80%) versus mock-infected (20%) mice. These data suggest that although exposure to a variety of pathogen challenges in the mouse model does not overtly impact cellular markers of immunity in aged female mice following de novo respiratory infection, subtle changes may emerge in other compartments or with increasing age.

## Introduction

Immune function declines with age (1–3). Because people are living longer, it is important to understand the impact of aging on their immune system so that vaccines and therapeutic strategies tailored for the elderly can be developed. The aging mouse model has many experimental advantages in terms of dissecting mechanisms underlying impaired immunity in the elderly. However, there are acknowledged limitations to the model (4–10). One important limitation is that mice are typically maintained under specific pathogen-free (SPF) conditions, whereas humans are exposed to a variety of pathogens throughout life. Accumulating evidence has demonstrated that microbial experience impacts the immune response, leading to the growing awareness that mouse husbandry has profound impacts on immunity (10–15). The immune system in the elderly is impacted profoundly by exposure to acute and chronic pathogens, and it has been suggested that the use of SPF mice with minimal exposure to pathogens makes mice a less relevant model for aging human immunity (5–7). Indeed, two landmark studies showed that whereas SPF mice had transcriptional profiles similar to human cord blood, Ag-experienced mice, induced either by serial pathogen infection (16) or by cohousing with pathogen-exposed pet store mice (17), had transcriptional profiles characteristic of adult humans. In addition, the Ag-experienced mice showed altered responses to vaccination against yellow fever (16) and enhanced immunity following infection with *Listeria monocytogenes* and *Plasmodium berghei* (17). These early studies involved the characterization of young adult mice. The present study’s goal was to extend these findings and determine the impact of early-life sequential Ag exposure on immunity in aged mice.

To generate immunologically experienced aged mice, we adapted the strategy of Reese et al. (16) by sequentially infecting young mice with four pathogens, including viruses and worms to establish acute and chronic infections, prior to aging the mice without any further challenges. Our data reveal that sequentially infected (SI) aged mice showed overall transcriptional activation of immune markers in peripheral blood, with some alterations in CD8 T cell phenotype compared with mock-infected (MI) aged mice, but that both SI and MI aged mice at 18–21 mo responded comparably to de novo H3N2 influenza and *Mycobacterium tuberculosis* infection, as assessed by survival, weight loss, and induction of epitope-specific T cells.

## Materials and Methods

### Mice, viruses, and infections

**FIGURE 1.**
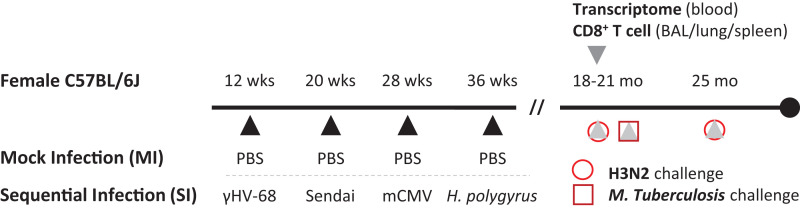
Sequential infection protocol and sample collection. Young (12-wk-old) female C57BL/6J mice were infected with γHV68, Sendai virus (Sendai), mCMV, and *H*. *polygyrus* at 8-wk intervals. Control mice were mock infected with PBS. SI mice were housed separately from MI mice. Cohorts of mice were maintained under SPF conditions until 18–25 mo of age. Mice aged to 18–20 mo were used for whole-blood transcriptomic analysis and for examination of CD8 T cell characteristics. Three other SI or MI cohorts were challenged with either the H3N2 influenza virus (two cohorts: 18–21 and 25 mo old) or *M. tuberculosis* (18–20 mo old).

Female C57BL/6, B6.SJL-Ptprca Pepcb/BoyJ (B6.CD45.1), and B6.129P2-Tcrb^tm1Mom^ Tcrd^tm1Mom^/J (TCRβδ^−/−^) mice were obtained from the Trudeau Institute animal facility or purchased from The Jackson Laboratory. All animal experiments were carefully evaluated and approved by the institutional animal care and use committee at Trudeau Institute. Twelve-wk-old female C57BL/6 mice were SI with 800 PFU murine γ-herpesvirus 68 (γHV68), 250 egg infectious dose 50 (EID_50_) Sendai virus (Enders strain), 2 × 10^4^ PFU murine CMV (mCMV), and 200 *Heligmosomoides polygyrus* larvae at 8-wk intervals. The γHV68 and Sendai viruses were administered via the intranasal route, mCMV was administered via i.p. injection, and *H. polygyrus* larvae were administered via oral gavage. Control mice were MI with PBS. SI and MI mice were housed in separate cages and aged to 18–25 mo under SPF conditions (Fig. 1). For the influenza challenge, MI and SI aged mice were infected with 3000 EID_50_ A/X-31 (H3N2) via the intranasal route. For the *M. tuberculosis* challenge, the H37Rv strain of *M. tuberculosis* was grown in Proskauer and Beck medium containing 0.05% Tween 80 to midlog phase and was preserved in aliquots at −70°C. MI and SI aged mice were aerosol infected with ∼100 CFU using a Glas-Col airborne infection system, as described previously (18). Bacteria in lung, mediastinal lymph node, liver, and spleen tissues were measured by counting viable CFU in homogenized tissue as described (18).

### Transcriptomic analysis

Blood was collected from six different cohorts of 18–20-mo-old MI and SI mice, and total RNA was extracted using the RiboPure RNA purification kit following the manufacturer’s instructions (Thermo Fisher Scientific, Waltham, MA). RNA quality and concentration were assessed by NanoDrop (Thermo Fisher Scientific) and the Agilent Bioanalyzer RNA 6000 Chip (Agilent, Santa Clara, CA). Total RNA (200 ng) was labeled, purified, and quantified using the Agilent Low-Input QuickAmp Labeling Kit according to the manufacturer’s recommendations. A total of 600 ng cyanine 3–labeled cRNA was hybridized for 17 h to Agilent SurePrint G3 Mouse Gene Expression v2 8 × 60K microarrays (G4852B). Following post-hybridization rinses, arrays were scanned using an Agilent SureScan microarray scanner at 3-μm resolution, and hybridization intensity data were extracted from the scanned images using the Agilent feature extraction software.

### Microarray data analysis

Raw microarray hybridization intensity data were log transformed to yield *z*-scores, and a *Z*-ratio (ZR) was calculated as the difference between the observed gene *z*-scores for the experimental and control comparisons and divided by the SD associated with the distribution of these differences. The false discovery rate (FDR) and *p* values were also calculated, both for individual cohorts (where possible) and for the combined set of SI and MI samples. Raw and *z*-score normalized gene expression data are deposited in the Gene Expression Omnibus (https://www.ncbi.nlm.nih.gov/geo/) repository under accession number GSE199730.

### Global expression changes

**FIGURE 2.**
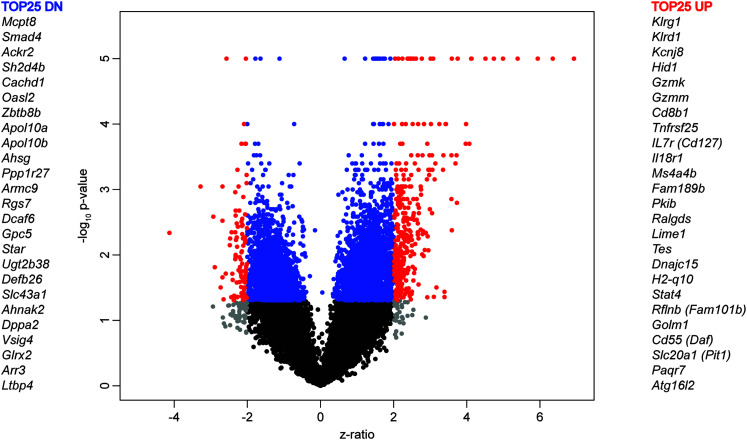
Volcano plot of microarray data. Gene expression comparisons for aged SI versus MI mice is shown in a volcano plot of ZR versus −log_10_ *p* value. Transcripts of absolute ZR ≥2 and *p* < 0.05 cutoffs, shown in red, illustrate the larger proportion of significantly upregulated than downregulated transcripts in the SI versus MI comparisons. Transcripts fulfilling only one of these criteria are shown in gray (|ZR| ≥2) or blue (*p* < 0.05). Transcripts labeled in black did not fulfill either selection criterion. Any *p* value of 10^−16^ and above is represented as 10^−5^ for the clarity of the figure.

A volcano plot (Fig. 2) of ZR versus −log_10_ *p* value illustrates a transcript-level comparison of aged SI/MI. Transcripts of absolute ZR ≥2 and *p* value <0.05 cutoffs are shown in red, with transcripts fulfilling only one of these criteria shown in gray (|ZR| ≥ 2) or blue (*p* < 0.05) and transcripts falling below these criteria labeled in black. Any *p* value of 10^−16^ or above is represented as 10^−5^ for clarity of the figure.

### Gene ontology enrichment analysis (ShinyGO version 0.61)

**FIGURE 3.**
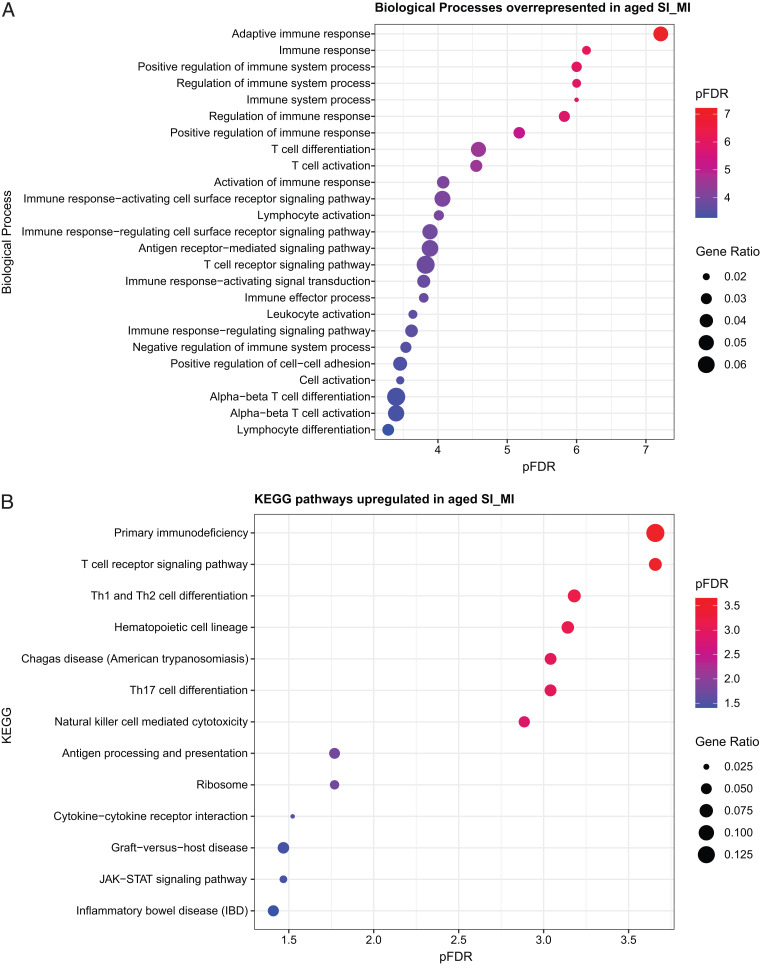
GO plots for upregulated transcripts. Using a list of 179 upregulated transcripts (157 unique) with an FDR ≤0.1 to query which top 25 GO biological processes (**A**) and KEGG pathways (**B**) were regulated (data analysis in ShinyGO version 0.1 and visualized using an RStudio-scripted dot plot).

Transcripts were included for gene ontology (GO) analysis if selected in a cohort (FDR, <0.05), and if the overall SI/MI comparison FDR was ≤0.1. This yielded a list of 179 upregulated transcripts (157 unique) to query which Top25 GO biological processes (Fig. 3A) and KEGG pathways (Fig. 3B) were regulated. No significant enrichment was found in either 43 downregulated transcripts with an FDR ≤0.1 or a list of 157 unique downregulated transcripts, sorted by lowest FDR. These data are summarized with fold change, ZR, and *p* values in Supplemental Table I. For both FDR and *p* values, values <10^−16^ are computationally returned as absolute 0 and are represented uniformly as 10^−5^.

### Lymphocyte isolation and flow cytometry

Mice were sacrificed at the indicated times, and their tissues were harvested. Cells from the bronchoalveolar lavage (BAL) were collected by lavage of the lungs five times with 1 ml HBSS without calcium and magnesium. Lung tissue was prepared by coarsely chopping the tissue followed by incubation in 0.5 mg/ml solution of collagenase D (Roche) and DNase (Sigma-Aldrich) for 30–45 min at 37°C. Single-cell suspensions were prepared from lung tissue, lymph nodes, or spleens by dispersing the tissues through a 70-mm nylon tissue strainer (BD Falcon). The cell suspensions were treated with buffered ammonium chloride solution to lyse erythrocytes. Lymphocytes were enriched from digested lung tissue by differential centrifugation, using a gradient of 40/80% Percoll (GE Healthcare).

Cell populations were incubated with Fc block (anti-CD16/32) for 15 min on ice, followed by staining with MHC class I or MHC class II tetramer reagents at room temperature for 1 h. Cells were then washed and stained with fluorochrome-labeled Abs to CD8, CD44, CD69, and CD127 (eBioscience); CD8, CD62L, and CD69 (BD Biosciences); CD43, CD62L, and CD4 (BioLegend); and KLRG1 (SouthernBiotech) for 30 min on ice. MHC class I peptide tetramers specific for γHV68 (ORF6_487_/D^b^), Sendai virus (NP_324_/K^b^), mCMV (M38_316_/D^b^), influenza virus (NP_366_/D^b^, PA_224_/D^b^, PB1_703_/K^b^, PB1-F2_62_/D^b^, and NS2_114_/K^b^), *M. tuberculosis* (Tb10.4_4-11_/K^b^), and an MHC class II peptide tetramer specific for *M. tuberculosis* (ESAT6_4-17_/A^b^) were generated by the Trudeau Institute Molecular Biology Core Facility (19). Stained samples were analyzed on a FACSCanto II or LSR II flow cytometer (BD Biosciences). Cells were gated on lymphocyte size and granularity, with doublets excluded. Data were analyzed with FlowJo software (BD Biosciences).

For analysis of resident memory T cells (Trm), intravital staining was performed immediately before mouse euthanasia and tissue harvest. To identify T cells resident in various tissues, 1.5 μg fluorophore-conjugated CD3e (145-2C11, BD Biosciences) Ab in 1× PBS was injected i.v.; 5 min after injection, mice were euthanized and exsanguinated prior to harvest of BAL, a negative control for intravital staining, and lungs. Tissues were processed and stained with MHC class I tetramers and fluorochrome-labeled Abs as described above.

### H. polygyrus imaging

Mice were euthanized via CO_2_ narcosis and exsanguination, and the peritoneum was opened to reveal the duodenum. A Nikon SMZ800 stereo zoom microscope was used to visualize the presence of the parasitic helminth, *H. polygyrus*, in the duodenum. The images were collected as TIFF files using a SPOT INSIGHT complementary metal oxide semiconductor camera and SPOT 5.3 software. Brightness and contrast were adjusted using Fiji software (ImageJ, National Institutes of Health).

### Repertoire analysis

Total memory (CD44^High^) CD8 T cells from the spleens of individual SI or MI aged mice were enriched by negative selection for CD8 cells using the BD Mouse CD8 T Lymphocyte Enrichment Kit, FACS sorted, and adoptively transferred into T cell–deficient βδ^−/−^ mice, along with CD8-depleted splenocytes from young, congenically disparate (B6.SJL-Ptprca Pepcb/BoyJ; B6.CD45.1) mice (5 × 10^6^ cells per recipient). CD8 memory cell numbers transferred were as follows: MI range, 5.6 × 10^5^ to 2.7 × 10^6^ (mean, 1.4 × 10^6^); SI range, 4.2 × 10^5^ to 3.3 × 10^6^ (mean, 1.6 × 10^6^). Recipient mice were challenged intranasally with X-31 influenza virus (H3N2, 3000 EID_50_). BAL and lung tissue were harvested at day 12 after infection, and the epitope specificity of the influenza-specific cells from individual SI and MI aged mice and control young mice was determined using tetramers NP_366_/D^b^, PA_224_/D^b^, PB1_703_/K^b^, PB1-F2_62_/D^b^, and NS2_114_/K^b^. Tissues were processed and stained with MHC class I tetramers and fluorochrome-labeled Abs as described above.

### Statistical analysis

Statistical analysis was performed with GraphPad Prism 5 software (GraphPad Software, La Jolla, CA). Differences were considered significant at *p* < 0.05.

## Results

### Generation of Ag-experienced aged mice

Previous murine studies on the impact of prior pathogen exposure on immunity have used a variety of models. These include pet store mice, mice that were cohoused with either wild or pet store mice, “wilded” mice (transfer of embryos from laboratory mice into pseudo-pregnant wild dams), “rewilded” mice (laboratory mice housed in an outdoor enclosure), inbred mice exposed to dirty bedding, mice to which natural microbiota had been transferred, or mice that had been serially infected with a variety of pathogens (12, 14–17, 20–24). For our studies, to more rigorously standardize the pathogens to which the mice were exposed, we chose to serially infect mice with specific pathogens, adapting a protocol developed by the Virgin laboratory using pathogens that elicit acute and chronic infections that included mouse γHV68, mCMV, influenza virus, and *H. polygyrus* (16). In our studies, we infected mice with Sendai virus, a mouse parainfluenza virus, instead of influenza virus so that we could monitor the response of the Ag-experienced mice after aging to de novo influenza infection (Fig. 1). The response of SI mice to a virus-specific tetramer after each sequential infection compared with naive mice is shown (Supplemental Fig. 1). This protocol induced a well-defined immune experience in mice prior to aging. The SI mice were subsequently aged until 18–25 mo of age before they were analyzed. Control mice were MI with PBS, aged separately, and are referred to as MI aged mice.

### Effects of sequential infection early in life on overall transcription in aged mice

Microarray analysis showed a significant and differential regulation of peripheral blood gene expression in SI versus MI mice aged to 20 mo. A transcript-level view is provided by the volcano plot of ZR change versus –log(*p* value) (Fig. 2), colorized to illustrate the significantly regulated genes in the SI aged mice. Many genes among the top 25 significantly upregulated genes represent immune response genes, including *Cd8b1*, *Gzmk*, *H2-Q10*, *Hcst*, *Klrd1*, *Klrg1*, *Il18r1*, and *Il7r*. This upregulation was also seen at the GO level, where 179 upregulated transcripts (157 unique genes) with an FDR ≤0.1 were queried to find the top 25 GO biological processes (Fig. 3A) and KEGG pathways (Fig. 3B). These almost exclusively included activation and regulation of many different innate and adaptive immune system processes and effectors, including T cell and proinflammatory disease states. In contrast, there was no significant enrichment in either 43 downregulated transcripts (FDR, ≤0.1) or in a list of 157 unique downregulated transcripts, sorted by lowest FDR.

### Phenotype of CD8 memory T cells at 18 mo of age

**FIGURE 4.**
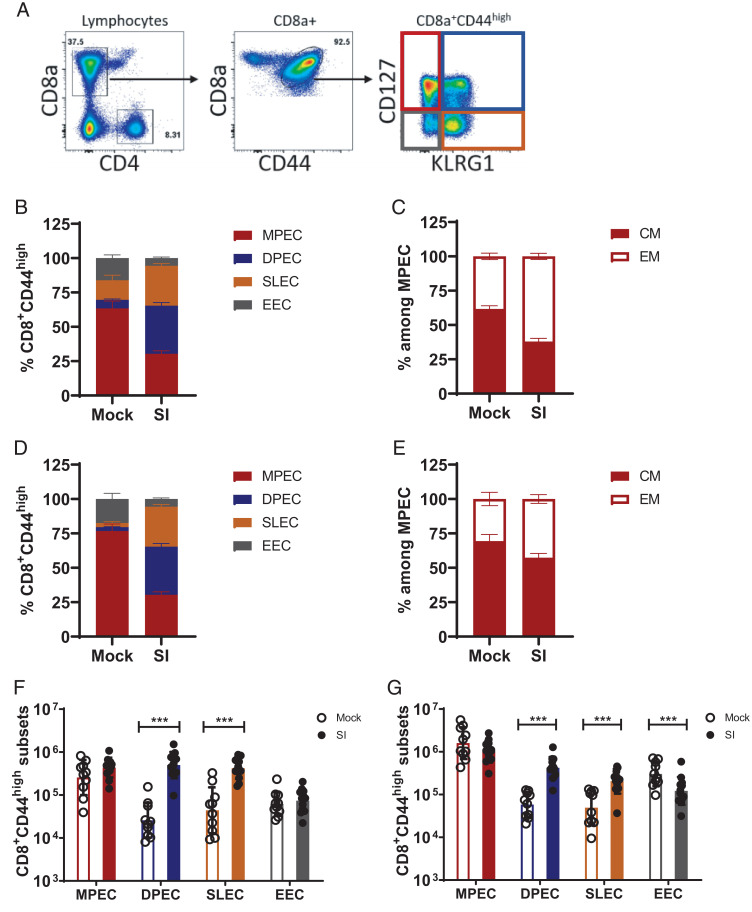
Distribution of peripheral CD8 T cells in MI and SI 18-mo-old mice. Lung and spleen cells from 18-mo-old MI and SI mice were analyzed for the expression of KLRG1 and CD127 to identify MPECs, DPECs, SLECs, and early effector (EEC) populations. (**A**) Representative flow cytometry staining illustrating the gating strategy. (**B**) Mean distribution of CD8 memory (CD8a^+^CD44^high^) subsets in the lungs of MI and SI mice. (**C**) Mean distribution of central memory (CM) and effector memory (EM) subsets within the MPEC population in the lung. (**D**) Mean distribution of CD8 memory subsets in the spleens of MI and SI mice. (**E**) Mean distribution of central memory (CM) and effector memory (EM) cells within the MPEC population in the spleen. (**F**) Absolute number of cells of each subset in the lungs of individual mice. (**G**) Absolute number of cells of each subset in the spleen of individual mice. Data are representative of two independent experiments and are presented as individual data points with bars representing mean ± SD. ***p < 0.0005 (two-tailed Student *t* test). *n* = 10–12 mice per group.

Following the resolution of infection, effector CD8 T cells develop into short-lived effector T cells (SLECs) or memory precursor effector T cells (MPECs) (25). Two phenotypic markers that have frequently been used to identify memory T cell subsets are IL-7R (CD127) and killer cell lectin-like receptor G1 (KLRG1) (26). Using these markers, memory T cells can be categorized into short-lived effector T cells (low CD127, high KLRG1, variably termed SLECs or Tsle) and memory precursor effector T cells (high CD127 and low KLRG1, variably termed MPECs or Tmpe). Double-positive cells, variably termed double-positive effector T cells (DPECs) or Tdpe, have been shown by fate-mapping analysis to lose expression of KLRG1 and develop into a subset of CD127-positive long-term memory cells (27, 28). In the present study, we examined the impact of sequential infection on T cell phenotype in aged mice. The gating strategy to identify the subsets of CD8 T cells is shown (Fig. 4A). The distributions of distinct memory T cell subsets in the lung (Fig. 4B, 4C, 4F) and spleen (Fig. 4D, 4E, 4G) of SI and MI aged mice are shown, based on frequency and absolute numbers of the various subsets of cells from the lung and spleen of individual mice. There were more SLECs in the SI aged mice, suggesting that much of the T cell compartment is chronically activated, presumably due to multiple chronic and latent infections (γHV68, mCMV, and *H. polygyrus*). The chronic state of activation is also supported by the finding that within the MPECs, the SI aged mice have a preponderance of effector memory cells, whereas the MI aged mice have an abundance of central memory cells. Note that the higher frequency of cells expressing KLRG1 (SLECs and DPECs) and IL7R/CD127 (DPECs) in the SI versus MI aged mice (Fig. 4B, 4D) is consistent with data showing that *Klrg1* and *IL7r/CD127* gene expression is upregulated in SI aged mice (Fig. 2).

### Distribution of circulating T cells and resident memory T cells in the lungs of sequentially infected and mock-infected aged mice

**FIGURE 5.**
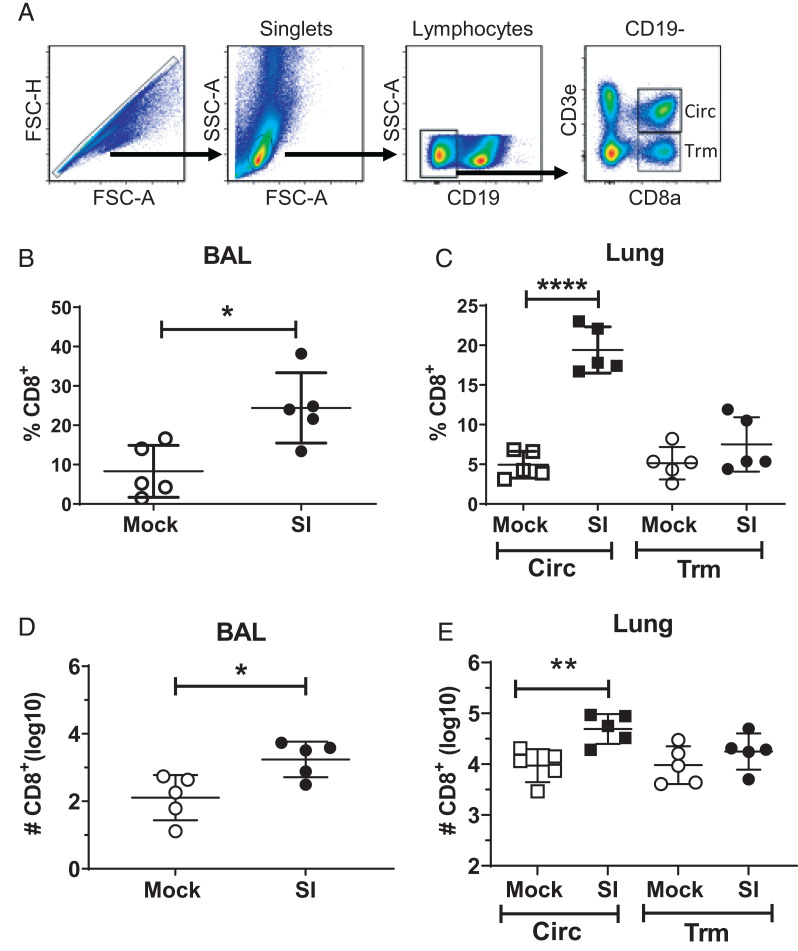
Identification of Trm in the lungs and spleens of SI and MI aged mice. (**A**) Gating strategy used to identify circulating cells and Trm after intravital labeling with anti-CD3e. (**B**) Frequencies of CD8 T cells in the BAL of SI and MI (Mock) 18-mo-old mice. (**C**) Frequencies of circulating cells (circ) and Trm in the lungs of SI and MI 18-mo-old mice. (**D**) The number of CD8 T cells in the BAL of SI and MI 18-mo-old mice. (**E**) The number of circulating cells and Trm in the lungs of SI and MI 18-mo-old mice. Circles represent Trm; squares represent circulating cells. Data are representative of two independent experiments and are presented as individual data points with bars representing mean ± SD; *n* = 5 mice per group. *p < 0.05, **p < 0.005, ****p < 0.0001 (two-tailed Student *t* test).

Memory T cells in mice can be divided into circulating T cell or Trm populations. Trm establish residence in tissues and serve as frontline responders to prevent reinfection (29). We enumerated CD8 T cells in the BAL and Trm and circulating CD8 T cells in the lung and spleen of SI and MI aged mice at 18 mo of age, using the gating strategy shown in Fig. 5A. There were statistically significant increased percentages and numbers of CD8 T cells in the BAL of SI aged mice (Fig. 5B, 5D); however, there were no statistically significant differences in numbers or frequency of Trm in the lungs of SI and MI aged mice (Fig. 5C, 5E). Although previous studies found that “dirty” pet store mice had increased numbers of Trm, our results differ and may reflect consequences of aging (17). We did, however, find enhanced frequencies and numbers of circulating cells in the lungs of SI aged mice (Fig. 5C, 5E).

### Distribution of virtual memory and true memory cells in sequentially and mock-infected aged mice

**FIGURE 6.**
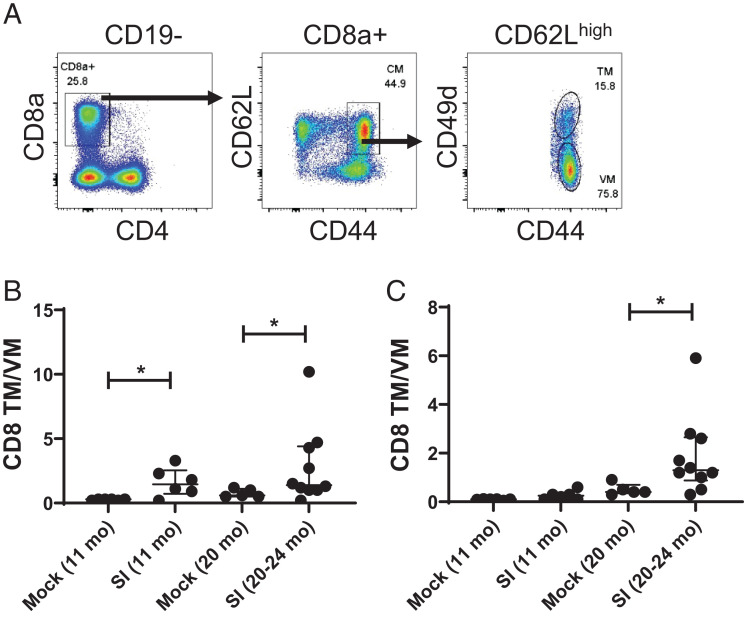
SI aged mice have a higher ratio of TM to VM CD8 T cells. Lungs and spleens from 11- and 20–24-mo-old SI and MI mice were analyzed for the ratio of CD8 central memory (CM) T cells of a VM and TM phenotype. (**A**) Flow cytometry gating strategy. (**B**) CD8 TM/VM ratio in lung. (**C**) CD8 TM/VM ratio in spleen. Data points represent individual mice with bars representing median and interquartile range. *p < 0.05 (Mann-Whitney *U* test). Data are pooled from two independent experiments; *n* = 6–10 mice per group.

Although memory cells are classically defined as a population of long-lasting Ag-specific T cells generated by exposure to Ag, a population of Ag-specific central memory cells has been identified in mice and humans (30–33) that develop in the absence of Ag and accumulate with age, termed virtual memory (VM) cells. It has been shown that VM cells represent the largest population of CD8 T cells in aged B6 mice (34). Consistent with this, we have previously shown a large population of VM cells in aged B6 mice that make a major contribution to the response of aged influenza-naive mice to influenza virus infection (35). Others have examined the impact of exposure to environmental Ags (36) and helminth infections (37, 38) on the population of VM cells, showing an increase in the population of VM cells due to Ag exposure. In the present studies, we investigated whether VM cells still dominated the response of Ag-experienced aged mice to de novo influenza infection. We used CD49 to distinguish classical “true” memory (TM; CD49d^high^) and VM (CD49d^low^) populations among CD44^high^CD62L^high^ central memory cells (Fig. 6A). The data show a higher ratio of TM/VM CD8 T cells in both the lung (Fig. 6B) and spleen (Fig. 6C) of SI 11-mo-old and 20–24-mo-old mice than in MI mice of the same age. This contrasts with the previous studies cited above showing accumulation of VM cells with age and Ag exposure and supports the conclusion that repeated infection biases the memory T cell pool to TM cells and diminishes the population of VM cells.

### The diversity of influenza virus epitopes recognized by CD8 memory T cells responding to de novo influenza infection is not profoundly affected by sequential infection of aged mice

**FIGURE 7.**
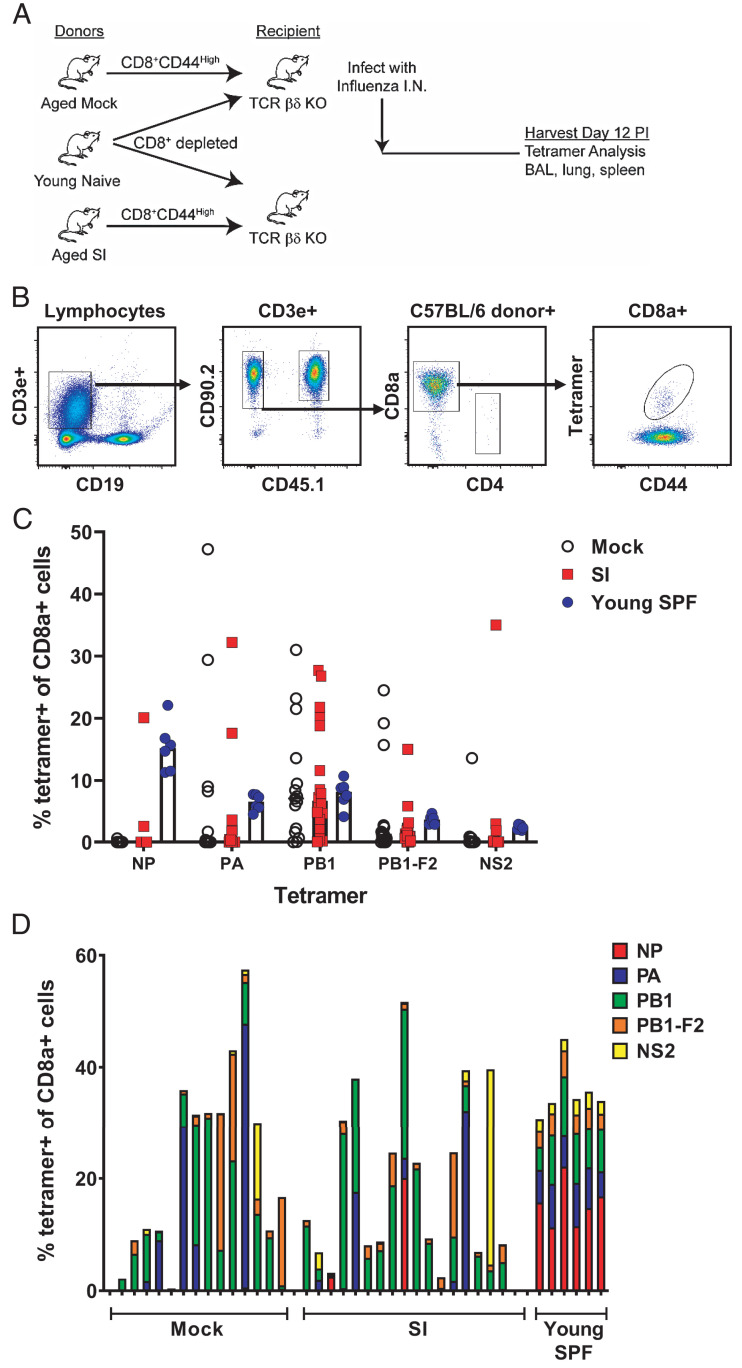
Impact of sequential infection on the CD8 T cell influenza-specific repertoire in aged mice. (**A**) Sorted CD44^high^ memory CD8 T cells from individual 18-mo-old MI and SI mice were transferred along with CD8 T cell–depleted splenocytes from CD45.1 congenic young mice into individual 2-mo-old T cell–deficient TCRβδ^−/−^ mice. Mice were intranasally infected with 3000 EID_50_ influenza A/X-31, and the responding CD8 T cells from BAL, lung, and spleen were analyzed for influenza tetramer specificity at 12 d after infection (PI). (**B**) Gating strategy to identify influenza tetramer-positive T cells after influenza infection, using a panel of five tetramers specific for NP, PA, PB1, PB1-F2, and NS2. Data points represent individual mice, with bars representing the median. (**C**) The frequency of tetramer-positive cells among CD8 T cells specific for each of the five epitopes in MI, SI, and young SPF (Y SPF) control mice. (**D**) Rainbow plots showing the distribution of tetramer-positive cells in individual MI and SI aged mice and young wild-type (WT) control mice. Bars represent individual mice, and colors within the bars represent specific epitopes, as shown in the key. Data are pooled from two independent experiments; mock, *n* = 15 mice; SI, *n* = 18 mice; Y SPF, *n* = 6 mice. KO, knockout.

As mice and humans age, the number of naive T cells decreases while memory T cell numbers increase. It has been suggested that this results in the inclusion of (cross-reactive) memory T cells into the response to new pathogens (39, 40). In support of this, we have previously shown that memory phenotype cells dominate the response to de novo influenza infection in aged animals (35). In addition, we have shown that the diversity of epitopes to which CD8 T cells from aged compared with young mice respond following influenza virus infection is dramatically reduced, particularly the frequency of CD8 T cells specific for the immunodominant influenza nucleoprotein (NP) epitope, NP_366_, in C57BL/6 mice (41). We attributed this to the decline in naive T cells associated with aging (42–46). In this study, we determined whether changes in the T cell makeup of SI aged mice, including the increased numbers of TM cells (as shown in Fig. 6), would impact the diversity of the epitope-specific response of aged mice to primary influenza virus infection. We transferred FACS-sorted memory (CD44^high^) CD8 T cells from influenza-naive SI and MI aged mice into T cell–deficient (βδ^−/−^) young mice. CD8 T cell–depleted splenocytes from young, congenic (B6.CD45.1) mice were cotransferred, and the mice were challenged with influenza virus (Fig. 7A). Responding transferred T cells from aged mice were identified, as shown in Fig. 7B. The responding T cells in individual mice were analyzed for repertoire diversity using T cell tetramers to five immunodominant influenza epitopes, described in the *Materials and methods* section, referred to as NP, PA, PB-1, PB1-F2, and NS2 (nonstructural protein 2) (Fig. 7C). The repertoire diversity within individual mice is presented as a rainbow plot (Fig. 7D), which shows the T cell repertoire in individual mice to be diverse and, as previously reported (41), showed a dramatic reduction in the frequency of CD8 T cells specific for NP in aged MI mice compared with young SPF mice (Fig. 7C, 7D). Furthermore, there was no evidence for a significant change in the frequency of NP-specific T cells in the SI aged mice (Fig. 7D).

### Sequentially infected and mock-infected aged mice respond comparably to de novo infection with influenza virus

**FIGURE 8.**
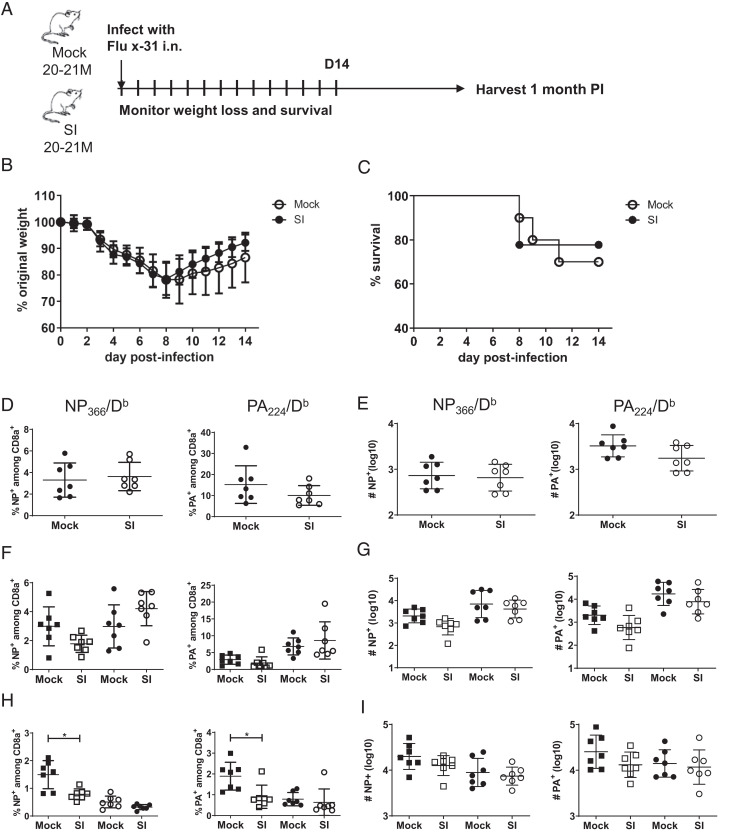
Response of MI and SI 20–21-mo-old mice to infection with influenza virus is comparable. (**A**) Experimental plan for influenza virus infection and monitoring of mice. MI and SI mice 20–21 mo old were intranasally infected with 3000 EID_50_ influenza A/X-31, then monitored for weight loss and survival, and their tissues were collected for tetramer staining at 1 mo after infection. (**B**) Weight loss. Data points represent mean with bars representing ±SD. (**C**) Survival was monitored for 14 d. Moribund mice were humanely euthanized. (**D**–**I**) The frequency and absolute numbers of T cells specific for NP- and PA-positive CD8 T cells in the BAL, lung, and spleen at 1 mo after infection are shown for individual mice. (D) The frequency of NP- and PA-positive T cells in the BAL. (E) The absolute number of NP- and PA-positive T cells in the BAL. (F) The frequency of NP- and PA-positive T cells in the lung. (G) The absolute number of NP- and PA-positive T cells in the lung. (H) The frequency of NP- and PA-specific T cells in the spleen. (I) The absolute number of NP- and PA-specific CD8 T cells in the spleen. Square symbols represent circulating cells, and circle symbols represent resident CD8a tetramer-positive populations. Data are representative of two independent experiments; *n* = 5–7 mice per group. *p < 0.05 (two-tailed Student *t* test).

SI and MI aged mice (20–21 mo) were infected with influenza virus, a pathogen they had not previously encountered, as shown in Fig. 8A. Weight loss after infection was comparable in the SI and MI aged mice, and the rate of recovery of weight was not statistically different between the two groups (Fig. 8B). Additionally, the percentage survival in the two groups was comparable in two independent experiments and was 70–80% at 14 d after infection (Fig. 8C). Analysis of T cells specific for two immunodominant epitopes, NP and PA, showed that there were no statistically significant differences in the percentage distribution of tetramer-positive T cells or in the absolute number of tetramer-positive T cells in the BAL (Fig. 8D, 8E), lung (Fig. 8F, 8G), or spleen (Fig. 8H, 8I) in two independent experiments. A third experiment, in which the mice were older when infected with influenza virus (25 mo compared with 20–21 mo) also showed similar weight loss between SI and MI aged mice. Importantly, however, 25-mo-old MI aged mice had a significantly lower survival rate (20%) than SI aged mice (80%), consistent with the role of Ag experience in affecting immunity of aged mice to a new respiratory pathogen (Supplemental Fig. 2). This is an intriguing result that needs to be confirmed in additional experiments with older mice.

### Sequentially infected and mock-infected aged mice respond comparably to de novo infection with *M. tuberculosis*

**FIGURE 9.**
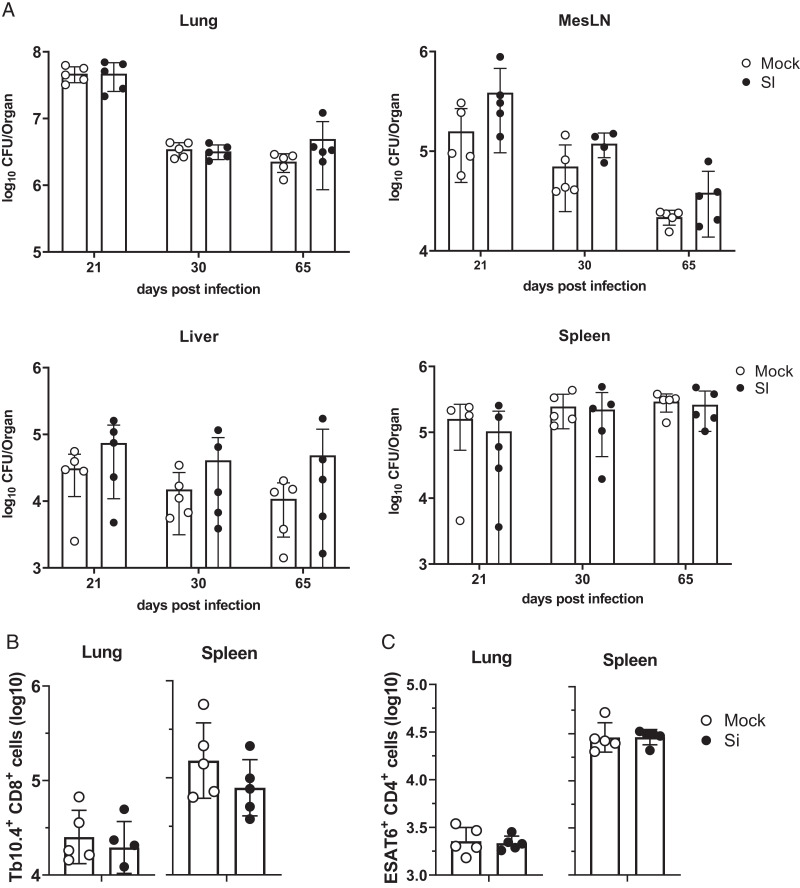
Response of MI and SI aged mice to *M. tuberculosis* infection is comparable. MI and SI mice were infected by aerosol with 100 CFU *M. tuberculosis* (Mtb). (**A**) Lung, mediastinal lymph node (MedLN), liver, and spleen were harvested at days 21, 30, and 65 after infection to evaluate bacterial burden. (**B**) Number of Tb10.4-specific CD8 T cells in the lung and spleen at day 21 after infection. (**C**) Number of ESAT6-specific CD4 T cells in the lung and spleen at day 21 after infection. Data are representative of two independent experiments; *n* = 5 mice per group.

SPF aged (18–20 mo) MI and SI mice were infected with *M. tuberculosis* and analyzed for bacterial load and responding T cells (Fig. 9). No statistical differences in bacterial load (assessed by CFU) were seen at days 21, 30, and 65 after infection in the lungs, mesenteric lymph nodes, livers, or spleens of the MI compared with SI mice (Fig. 9A). Likewise, no statistical differences were seen in the numbers of tetramer-positive Tb10.4-specific CD8 T cells (Fig. 9B) or ESAT-6-specific CD4 T cells (Fig. 9C) assessed with Ag-specific tetramers.

## Discussion

Our results show a clear effect of sequential infection on the transcriptional profile in peripheral blood from aged (18–20 mo) mice, reflecting overall activation, particularly of genes associated with T cell function and immunity. Despite this, we identified only subtle changes in T cell phenotype, including a dominance of SLECs in SI mice and increased numbers of effector memory cells compared with central memory cells in the memory (MPEC) population. Both are probably a consequence of continual stimulation by the chronic and latent infections by the pathogens used for the sequential infection. There was also no increase in the numbers of Trm in the spleens and lungs of SI aged mice. Rather, the increased numbers of CD8 T cells in these organs were circulating, rather than resident, T cells. One clear impact of sequential infection was the increased ratio of TM to VM CD8 T cells. However, there was no discernible impact of sequential infection on the repertoire diversity of CD8 T cells responding to immunodominant influenza virus epitopes, nor was there an impact of sequential infection on the response of aged mice to primary infection with influenza virus or *M. tuberculosis* at 18–21 mo of age.

These data contribute an aging perspective to the literature comparing the immune response of “clean,” SPF-maintained laboratory mice and “dirty” mice, generated by a variety of methods, including sequential infection, cohousing with pet store or wild mice, or transfer of microbiota from wild mice (14–17, 20–24, 47). The published literature on immunity in dirty mice has shown variable results, with some studies showing enhanced immunity (17, 47, 48) and others showing depressed immunity (15, 49) or no effect (48). Variations in these results likely reflect differences in the manner of inducing antigenic experience, the pathogen used to assess immunity, the type of immune responses analyzed, and the time points and/or age at which the assessments were undertaken.

The most important distinction in our studies from those previously published is that we evaluated the difference between clean and dirty aged mice to reflect the immune experiences that accumulate in elderly humans. An important variable for our studies may be the age at which the mice were analyzed. With the exception of the data presented in Supplemental Fig. 2, all of our analysis was performed on SI or MI mice that had been aged for 18–21 mo. This age in C57BL/6 mice corresponds to a human age of 56–69 y and is a typical age of mice used for aging studies (50). When 18–21-mo-old mice were challenged with influenza virus or *M. tuberculosis* in this study, there was no discernible difference in the response of MI and SI aged mice. However, in a single experiment in which we challenged 25-mo-old MI and SI mice with influenza virus, the SI aged mice exhibited a significantly enhanced protection of 80% survival rate versus 20% in MI aged mice. The finding that sequential infection protects older mice at 25 mo of age from influenza infection would need to be confirmed with additional studies, and characterization of the other immune parameters described in this study would need to be repeated with mice 25 mo of age or older. These studies are beyond the scope of the present article. However, 18–21-mo-old mice are typically used for aging studies, and it is unclear why we would only see an effect in 25-mo-old mice.

What is the significance of our findings for the validity of SI aged mice as models for elderly humans? There are two possibilities. One is that, even though the aged mice are maintained in SPF conditions, they are not germ-free, so there is still exposure over time to antigenic stimulation. Perhaps this exposure is sufficient to represent infections throughout life and it is not necessary to deliberately infect mice as they age to mimic the human situation. However, transcriptional analysis showed that the SI aged mice were more generally activated, and CD8 T cells differed subtly in their CD8 T cell phenotype, indicating that sequential infection caused immune activation beyond that of SPF-maintained mice. This might be a consequence of the fact that the sequential infection included chronic pathogens. A second possibility, consistent with studies in young “dirty” mice, is that there is little effect of antigenic history on the primary immune response to influenza virus, as shown in one (15, 49) but not all (47) studies. Because an effect of prior Ag exposure was seen on vaccination, resulting in compromised responses to heterosubtypic challenge of young mice (15), additional studies in “dirty” aged mice are needed to assess the innate, humoral, and cellular responses to primary infection and vaccination.

Our experiments were designed to test whether Ag experience would impact the T cell repertoire in aged mice. It has been shown that there is a dramatic reduction of the naive T cell repertoire with aging in mice and humans (42, 51–54). We previously found a dramatic decline in the proportion of naive T cells in aged mice that resulted in a “hole in the repertoire,” manifested by the reduced response of CD8 T cells specific for a normally immunodominant influenza virus epitope in the influenza NP, NP_366-374_ (41). We have hypothesized that because of this decline in naive T cell repertoire diversity, responses to new Ags would be significantly mediated by memory T cells generated during the lifespan that are fortuitously cross-reactive with the new Ags (39). In support of this hypothesis, our previous data using dual adoptive transfer of memory and naive CD8 T cells from influenza-naive aged mice showed that aged memory CD8 T cells outcompete the response of aged naive CD8 T cells to a de novo influenza virus infection and further that these cells were of the VM phenotype rather than TM T cells (35). In this study, we demonstrate a clear shift in the memory CD8 T cell phenotype in SI aged mice to a TM phenotype. Despite this, and in contrast to evidence showing that specific priming with influenza virus early in life enhanced the anti-NP response of aged mice (55), memory CD8 T cells from SI aged mice did not reconstitute the strong response to influenza NP that is characteristic of young mice. This suggests that the memory repertoire generated by sequential infection cannot compensate for the diversity of the naive repertoire in young mice.

Prior infection with pathogens elicits a variety of Ag-specific immune responses and induces generalized activation, inflammation, and broad nonspecific effects on innate immunity (48, 56–58), and our data suggest that neither Ag-specific nor nonspecific effects of sequential infection were sufficient to overtly change the course of primary immunity to influenza virus or *M. tuberculosis*.

We chose to make our mice “dirty” by sequential infection rather than cohousing with pet store mice to control the type and duration of pathogen exposure. We essentially followed a previous protocol and sequentially infected our mice with four distinct pathogens (16), although practical considerations caused us to change the order of infection slightly and to substitute Sendai virus for influenza virus. We acknowledge that the order of infection may have an impact, but these studies are beyond the scope of the present study. Importantly, we monitored the response to each subsequent infection in SI mice compared with naive mice to confirm adequate infection in all cases. An obvious limitation of this approach is the small number of pathogens used. A better strategy may be to transfer the pooled microbiome of pet store or wild-caught mice to laboratory mice prior to aging, because several studies have underscored the importance of the microbiome in response to infection. For example, studies in which antibiotics disrupted the microbiome demonstrated the important role of the microbiome in regulating cellular and humoral immunity to influenza virus (59). More recently, transfer of microbiota from wild mice into laboratory mice was shown to greatly enhance survival to an otherwise lethal infection with heterosubtypic influenza virus (47). Another study failed to find an impact on primary influenza virus infection but described a depressed response after heterosubtypic influenza challenge (15). Transferring microbiota from wild mice into genetically defined laboratory mice prior to aging would combine the advantages of well-defined genetics of inbred mice with identical microbiota reflecting enhanced Ag experience.

Our studies are the first to assess the impact of Ag experience in the aging mouse model. The goal was to develop a “better” aging mouse model, reflective of antigenic experience characteristic of aged humans. Our key finding was that sequential infection did not have a profound impact on primary immunity in aged mice in response to de novo influenza or *M. tuberculosis* infections in terms of weight loss, survival, or the numbers of tetramer-positive CD8 T cells elicited. Clearly, more extensive studies are necessary, including the generation of “dirty” aged mice by different means, assessing immunity at different ages, and more rigorously assessing immunity in both the adaptive and innate immune systems.

## Supplementary Material

Supplemental 1 (PDF)

Supplemental 2 (XLSX)

## References

[1] McElhaney, J. E., R. B. Effros. 2009. Immunosenescence: what does it mean to health outcomes in older adults? Curr. Opin. Immunol. 21: 418–424. 19570667 10.1016/j.coi.2009.05.023 PMC2725188

[2] Gruver, A. L., L. L. Hudson, G. D. Sempowski. 2007. Immunosenescence of ageing. J. Pathol. 211: 144–156. 17200946 10.1002/path.2104 PMC1931833

[3] Grubeck-Loebenstein, B., G. Wick. 2002. The aging of the immune system. Adv. Immunol. 80: 243–284. 12078483 10.1016/s0065-2776(02)80017-7

[4] Mestas, J., C. C. Hughes. 2004. Of mice and not men: differences between mouse and human immunology. J. Immunol. 172: 2731–2738. 14978070 10.4049/jimmunol.172.5.2731

[5] Virgin, H. W., E. J. Wherry, R. Ahmed. 2009. Redefining chronic viral infection. Cell 138: 30–50. 19596234 10.1016/j.cell.2009.06.036

[6] Davis, M. M. 2012. Immunology taught by humans. Sci. Transl. Med. 4: 117fs2. 10.1126/scitranslmed.3003385 PMC3762495 22261029

[7] Goronzy, J. J., C. M. Weyand. 2013. Understanding immunosenescence to improve responses to vaccines. Nat. Immunol. 14: 428–436. 23598398 10.1038/ni.2588 PMC4183346

[8] Seok, J., H. S. Warren, A. G. Cuenca, M. N. Mindrinos, H. V. Baker, W. Xu, D. R. Richards, G. P. McDonald-Smith, H. Gao, L. Hennessy, Inflammation and Host Response to Injury, Large Scale Collaborative Research Program. 2013. Genomic responses in mouse models poorly mimic human inflammatory diseases. Proc. Natl. Acad. Sci. USA 110: 3507–3512. 23401516 10.1073/pnas.1222878110 PMC3587220

[9] Takao, K., T. Miyakawa. 2015. Genomic responses in mouse models greatly mimic human inflammatory diseases. [Published erratum appears in 2015 *Proc. Natl. Acad. Sci. USA* 112: E1163–E1167.] Proc. Natl. Acad. Sci. USA 112: 1167–1172. 25092317 10.1073/pnas.1401965111 PMC4313832

[10] Masopust, D., C. P. Sivula, S. C. Jameson. 2017. Of mice, dirty mice, and men: using mice to understand human immunology. J. Immunol. 199: 383–388. 28696328 10.4049/jimmunol.1700453 PMC5512602

[11] Willyard, C. 2018. Squeaky clean mice could be ruining research. Nature 556: 16–18. 29620765 10.1038/d41586-018-03916-9

[12] Kuypers, M., T. Despot, T. Mallevaey. 2021. Dirty mice join the immunologist’s toolkit. Microbes Infect. 23: 104817. 33785421 10.1016/j.micinf.2021.104817

[13] Ericsson, A. C., D. R. Montonye, C. R. Smith, C. L. Franklin. 2017. Modeling a superorganism – considerations regarding the use of “dirty” mice in biomedical research. Yale J. Biol. Med. 90: 361–371. 28955177 PMC5612181

[14] Hamilton, S. E., V. P. Badovinac, L. K. Beura, M. Pierson, S. C. Jameson, D. Masopust, T. S. Griffith. 2020. New insights into the immune system using dirty mice. J. Immunol. 205: 3–11. 32571979 10.4049/jimmunol.2000171 PMC7316151

[15] Fiege, J. K., K. E. Block, M. J. Pierson, H. Nanda, F. K. Shepherd, C. K. Mickelson, J. M. Stolley, W. E. Matchett, S. Wijeyesinghe, D. K. Meyerholz, . 2021. Mice with diverse microbial exposure histories as a model for preclinical vaccine testing. Cell Host Microbe 29: 1815–1827.e6. 34731647 10.1016/j.chom.2021.10.001 PMC8665115

[16] Reese, T. A., K. Bi, A. Kambal, A. Filali-Mouhim, L. K. Beura, M. C. Bürger, B. Pulendran, R. P. Sekaly, S. C. Jameson, D. Masopust, . 2016. Sequential infection with common pathogens promotes human-like immune gene expression and altered vaccine response. Cell Host Microbe 19: 713–719. 27107939 10.1016/j.chom.2016.04.003 PMC4896745

[17] Beura, L. K., S. E. Hamilton, K. Bi, J. M. Schenkel, O. A. Odumade, K. A. Casey, E. A. Thompson, K. A. Fraser, P. C. Rosato, A. Filali-Mouhim, . 2016. Normalizing the environment recapitulates adult human immune traits in laboratory mice. Nature 532: 512–516. 27096360 10.1038/nature17655 PMC4871315

[18] Roberts, A., A. Cooper, J. Belisle, M. Turner, M. Gonzalez-Juarerro, I. M. Orme. 2002. Murine models of tuberculosis. In Methods in Microbiology. S. Kaufmann, D. Kabelitz, eds. Academic Press, London, p. 433–462.

[19] Reiley, W. W., S. T. Wittmer, L. M. Ryan, S. M. Eaton, L. Haynes, G. M. Winslow, D. L. Woodland. 2012. Maintenance of peripheral T cell responses during *Mycobacterium tuberculosis* infection. J. Immunol. 189: 4451–4458. 23028057 10.4049/jimmunol.1201153 PMC3819137

[20] Huggins, M. A., F. V. Sjaastad, M. Pierson, T. A. Kucaba, W. Swanson, C. Staley, A. R. Weingarden, I. J. Jensen, D. B. Danahy, V. P. Badovinac, . 2019. Microbial exposure enhances immunity to pathogens recognized by TLR2 but increases susceptibility to cytokine storm through TLR4 sensitization. Cell Rep. 28: 1729–1743.e5. 31412243 10.1016/j.celrep.2019.07.028 PMC6703181

[21] Rosshart, S. P., J. Herz, B. G. Vassallo, A. Hunter, M. K. Wall, J. H. Badger, J. A. McCulloch, D. G. Anastasakis, A. A. Sarshad, I. Leonardi, . 2019. Laboratory mice born to wild mice have natural microbiota and model human immune responses. Science 365: eaaw4361. 31371577 10.1126/science.aaw4361 PMC7377314

[22] Leung, J. M., S. A. Budischak, H. Chung The, C. Hansen, R. Bowcutt, R. Neill, M. Shellman, P. Loke, A. L. Graham. 2018. Rapid environmental effects on gut nematode susceptibility in rewilded mice. PLoS Biol. 16: e2004108. 29518091 10.1371/journal.pbio.2004108 PMC5843147

[23] Lin, J. D., J. C. Devlin, F. Yeung, C. McCauley, J. M. Leung, Y. H. Chen, A. Cronkite, C. Hansen, C. Drake-Dunn, K. V. Ruggles, . 2020. Rewilding Nod2 and Atg16l1 mutant mice uncovers genetic and environmental contributions to microbial responses and immune cell composition. Cell Host Microbe 27: 830–840.e4. 32209431 10.1016/j.chom.2020.03.001 PMC7228860

[24] Yeung, F., Y. H. Chen, J. D. Lin, J. M. Leung, C. McCauley, J. C. Devlin, C. Hansen, A. Cronkite, Z. Stephens, C. Drake-Dunn, . 2020. Altered immunity of laboratory mice in the natural environment is associated with fungal colonization. Cell Host Microbe 27: 809–822.e6. 32209432 10.1016/j.chom.2020.02.015 PMC7276265

[25] Sallusto, F., D. Lenig, R. Förster, M. Lipp, A. Lanzavecchia. 1999. Two subsets of memory T lymphocytes with distinct homing potentials and effector functions. Nature 401: 708–712. 10537110 10.1038/44385

[26] Sallusto, F., J. Geginat, A. Lanzavecchia. 2004. Central memory and effector memory T cell subsets: function, generation, and maintenance. Annu. Rev. Immunol. 22: 745–763. 15032595 10.1146/annurev.immunol.22.012703.104702

[27] Joshi, N. S., W. Cui, A. Chandele, H. K. Lee, D. R. Urso, J. Hagman, L. Gapin, S. M. Kaech. 2007. Inflammation directs memory precursor and short-lived effector CD8^+^ T cell fates via the graded expression of T-bet transcription factor. Immunity 27: 281–295. 17723218 10.1016/j.immuni.2007.07.010 PMC2034442

[28] Herndler-Brandstetter, D., H. Ishigame, R. Shinnakasu, V. Plajer, C. Stecher, J. Zhao, M. Lietzenmayer, L. Kroehling, A. Takumi, K. Kometani, . 2018. KLRG1^+^ effector CD8^+^ T cells lose KLRG1, differentiate into all memory T cell lineages, and convey enhanced protective immunity. Immunity 48: 716–729.e8. 29625895 10.1016/j.immuni.2018.03.015 PMC6465538

[29] Masopust, D., V. Vezys, A. L. Marzo, L. Lefrançois. 2001. Preferential localization of effector memory cells in nonlymphoid tissue. Science 291: 2413–2417. 11264538 10.1126/science.1058867

[30] White, J. T., E. W. Cross, M. A. Burchill, T. Danhorn, M. D. McCarter, H. R. Rosen, B. O’Connor, R. M. Kedl. 2016. Virtual memory T cells develop and mediate bystander protective immunity in an IL-15-dependent manner. Nat. Commun. 7: 11291. 27097762 10.1038/ncomms11291 PMC4844673

[31] White, J. T., E. W. Cross, R. M. Kedl. 2017. Antigen-inexperienced memory CD8^+^ T cells: where they come from and why we need them. Nat. Rev. Immunol. 17: 391–400. 28480897 10.1038/nri.2017.34 PMC5569888

[32] Lee, J. Y., S. E. Hamilton, A. D. Akue, K. A. Hogquist, S. C. Jameson. 2013. Virtual memory CD8 T cells display unique functional properties. Proc. Natl. Acad. Sci. USA 110: 13498–13503. 23898211 10.1073/pnas.1307572110 PMC3746847

[33] Jacomet, F., E. Cayssials, S. Basbous, A. Levescot, N. Piccirilli, D. Desmier, A. Robin, A. Barra, C. Giraud, F. Guilhot, . 2015. Evidence for eomesodermin-expressing innate-like CD8^+^ KIR/NKG2A^+^ T cells in human adults and cord blood samples. Eur. J. Immunol. 45: 1926–1933. 25903796 10.1002/eji.201545539

[34] Chiu, B. C., B. E. Martin, V. R. Stolberg, S. W. Chensue. 2013. Cutting edge: Central memory CD8 T cells in aged mice are virtual memory cells. J. Immunol. 191: 5793–5796. 24227783 10.4049/jimmunol.1302509 PMC3858473

[35] Lanzer, K. G., T. Cookenham, W. W. Reiley, M. A. Blackman. 2018. Correction to: Virtual memory cells make a major contribution to the response of aged influenza-naïve mice to influenza virus infection. Immun. Ageing 15: 18. 30093911 10.1186/s12979-018-0122-y PMC6081820

[36] Moudra, A., V. Niederlova, J. Novotny, L. Schmiedova, J. Kubovciak, T. Matejkova, A. Drobek, M. Pribikova, R. Stopkova, D. Cizkova, . 2021. Phenotypic and clonal stability of antigen-inexperienced memory-like T cells across the genetic background, hygienic status, and aging. J. Immunol. 206: 2109–2121. 33858960 10.4049/jimmunol.2001028 PMC7610663

[37] Lin, J. S., K. Mohrs, F. M. Szaba, L. W. Kummer, E. A. Leadbetter, M. Mohrs. 2019. Virtual memory CD8 T cells expanded by helminth infection confer broad protection against bacterial infection. Mucosal Immunol. 12: 258–264. 30361537 10.1038/s41385-018-0100-x PMC6301144

[38] Rolot, M., A. M. Dougall, A. Chetty, J. Javaux, T. Chen, X. Xiao, B. Machiels, M. E. Selkirk, R. M. Maizels, C. Hokke, . 2018. Helminth-induced IL-4 expands bystander memory CD8^+^ T cells for early control of viral infection. Nat. Commun. 9: 4516–4532. 30375396 10.1038/s41467-018-06978-5 PMC6207712

[39] Woodland, D. L., M. A. Blackman. 2006. Immunity and age: living in the past? Trends Immunol. 27: 303–307. 16731040 10.1016/j.it.2006.05.002 PMC7185388

[40] Blackman, M. A., D. L. Woodland. 2011. The narrowing of the CD8 T cell repertoire in old age. Curr. Opin. Immunol. 23: 537–542. 21652194 10.1016/j.coi.2011.05.005 PMC3163762

[41] Yager, E. J., M. Ahmed, K. Lanzer, T. D. Randall, D. L. Woodland, M. A. Blackman. 2008. Age-associated decline in T cell repertoire diversity leads to holes in the repertoire and impaired immunity to influenza virus. J. Exp. Med. 205: 711–723. 18332179 10.1084/jem.20071140 PMC2275391

[42] Naylor, K., G. Li, A. N. Vallejo, W. W. Lee, K. Koetz, E. Bryl, J. Witkowski, J. Fulbright, C. M. Weyand, J. J. Goronzy. 2005. The influence of age on T cell generation and TCR diversity. J. Immunol. 174: 7446–7452. 15905594 10.4049/jimmunol.174.11.7446

[43] Hale, J. S., T. E. Boursalian, G. L. Turk, P. J. Fink. 2006. Thymic output in aged mice. Proc. Natl. Acad. Sci. USA 103: 8447–8452. 16717190 10.1073/pnas.0601040103 PMC1482512

[44] Čičin-Šain, L., I. Messaoudi, B. Park, N. Currier, S. Planer, M. Fischer, S. Tackitt, D. Nikolich-Zugich, A. Legasse, M. K. Axthelm, . 2007. Dramatic increase in naive T cell turnover is linked to loss of naive T cells from old primates. Proc. Natl. Acad. Sci. USA 104: 19960–19965. 18056811 10.1073/pnas.0705905104 PMC2148405

[45] Ahmed, M., K. G. Lanzer, E. J. Yager, P. S. Adams, L. L. Johnson, M. A. Blackman. 2009. Clonal expansions and loss of receptor diversity in the naive CD8 T cell repertoire of aged mice. J. Immunol. 182: 784–792. 19124721 10.4049/jimmunol.182.2.784 PMC2724652

[46] Čičin-Šain, L., S. Smyk-Pearson, N. Currier, L. Byrd, C. Koudelka, T. Robinson, G. Swarbrick, S. Tackitt, A. Legasse, M. Fischer, . 2010. Loss of naive T cells and repertoire constriction predict poor response to vaccination in old primates. J. Immunol. 184: 6739–6745. 20483749 10.4049/jimmunol.0904193 PMC3504654

[47] Rosshart, S. P., B. G. Vassallo, D. Angeletti, D. S. Hutchinson, A. P. Morgan, K. Takeda, H. D. Hickman, J. A. McCulloch, J. H. Badger, N. J. Ajami, . 2017. Wild mouse gut microbiota promotes host fitness and improves disease resistance. Cell 171: 1015–1028.e13. 29056339 10.1016/j.cell.2017.09.016 PMC6887100

[48] Labuda, J. C., K. D. Fong, S. J. McSorley. 2022. Cohousing with dirty mice increases the frequency of memory T cells and has variable effects on intracellular bacterial infection. Immunohorizons 6: 184–190. 35210292 10.4049/immunohorizons.2100069 PMC9624231

[49] Coughlan, L. 2021. Caught in a trap: How pre-clinical studies in laboratory mice exaggerate vaccine responses. Cell. Rep. Med. 2: 100484. 35028623 10.1016/j.xcrm.2021.100484 PMC8715072

[50] Flurkey, K., J. M. Currer, D. E. Harrison. 2007. The mouse in aging research. In The Mouse in Biomedical Research: History, Wild Mice, and Genetics, 2nd Ed. J. G. Fox, ed. American College Laboratory Animal Medicine (Elsevier), Burlington, MA. p. 637–672.

[51] Mosley, R. L., M. M. Koker, R. A. Miller. 1998. Idiosyncratic alterations of TCR size distributions affecting both CD4 and CD8 T cell subsets in aging mice. Cell. Immunol. 189: 10–18. 9758689 10.1006/cimm.1998.1369

[52] Messaoudi, I., J. Lemaoult, J. A. Guevara-Patino, B. M. Metzner, J. Nikolich-Zugich. 2004. Age-related CD8 T cell clonal expansions constrict CD8 T cell repertoire and have the potential to impair immune defense. J. Exp. Med. 200: 1347–1358. 15545358 10.1084/jem.20040437 PMC2211915

[53] Nikolich-Žugich, J. 2005. T cell aging: naive but not young. J. Exp. Med. 201: 837–840. 15781575 10.1084/jem.20050341 PMC2213096

[54] Fagnoni, F. F., R. Vescovini, G. Passeri, G. Bologna, M. Pedrazzoni, G. Lavagetto, A. Casti, C. Franceschi, M. Passeri, P. Sansoni. 2000. Shortage of circulating naive CD8^+^ T cells provides new insights on immunodeficiency in aging. Blood 95: 2860–2868. 10779432

[55] Valkenburg, S. A., V. Venturi, T. H. Dang, N. L. Bird, P. C. Doherty, S. J. Turner, M. P. Davenport, K. Kedzierska. 2012. Early priming minimizes the age-related immune compromise of CD8^+^ T cell diversity and function. [Published erratum appears in 2012 *PLoS Pathog*. 8: 10.1371/annotation/e142f9de-7f30-4759-bda1-a651e86d5ba6.] PLoS Pathog. 8: e1002544. 22383879 10.1371/journal.ppat.1002544 PMC3285595

[56] Hooper, L. V., D. R. Littman, A. J. Macpherson. 2012. Interactions between the microbiota and the immune system. Science 336: 1268–1273. 22674334 10.1126/science.1223490 PMC4420145

[57] Honda, K., D. R. Littman. 2012. The microbiome in infectious disease and inflammation. Annu. Rev. Immunol. 30: 759–795. 22224764 10.1146/annurev-immunol-020711-074937 PMC4426968

[58] Song, W. M., M. Colonna. 2018. Immune Training Unlocks Innate Potential. Cell 172: 3–5. 29328917 10.1016/j.cell.2017.12.034

[59] Ichinohe, T., I. K. Pang, Y. Kumamoto, D. R. Peaper, J. H. Ho, T. S. Murray, A. Iwasaki. 2011. Microbiota regulates immune defense against respiratory tract influenza A virus infection. Proc. Natl. Acad. Sci. USA 108: 5354–5359. 21402903 10.1073/pnas.1019378108 PMC3069176

