# Supplementary material for: Sequential Early-Life Infections Alter Peripheral Blood Transcriptomics in Aging Female Mice but Not the Response to De Novo Infection with Influenza Virus or M. tuberculosis
Source: Immunohorizons. 2023 Aug 9;7(8):562–76. doi: 10.4049/immunohorizons.2200066 (PMC10587504; doi:10.4049/immunohorizons.2200066)
Supplement: Supplemental 1 (PDF) [file IH_2200066_Supplemental_1.pdf]

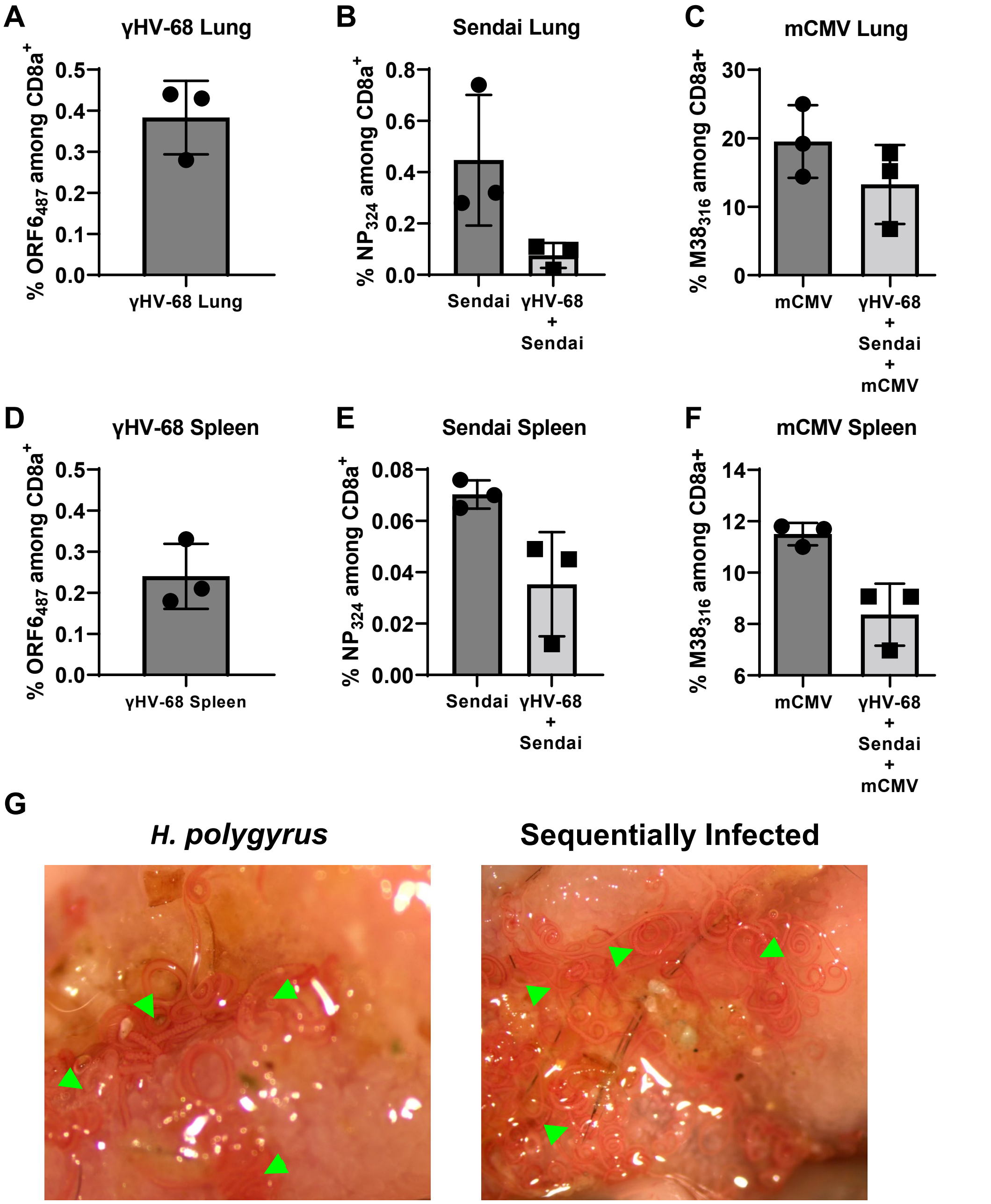

Supplemental Figure 1. Response of naïve and sequentially infected mice to each pathogen. 12-week-old SPF C57BL/6 mice were sequentially infected with γHV-68, Sendai, mCMV and *H. polygyrus*. Naïve mice were also infected with individual pathogens to serve as controls. For viral infections, lungs and spleens were collected at 1-month post-infection and stained for tetramer-specific CD8 T cells. A, D. Frequency of γHV-68 $ORF6_{487}$ CD8 T cells in the lungs and spleens of mice infected with γHV-68. B, E. Frequency of Sendai NP_{324} CD8 T cells in lungs and spleens of mice infected with Sendai alone or γHV-68 followed by Sendai. C, F. Frequency of mCMV $M38_{316}$ CD8 T cells in lungs and spleens of mice infected with mCMV alone or sequentially with γHV-68, Sendai and mCMV. G. Bright-field images of *H. polygyrus* worms (green arrows) in the duodenum of mice infected with *H. polygyrus* alone or sequentially infected with γHV-68, Sendai and mCMV followed by *H. polygyrus* infection.

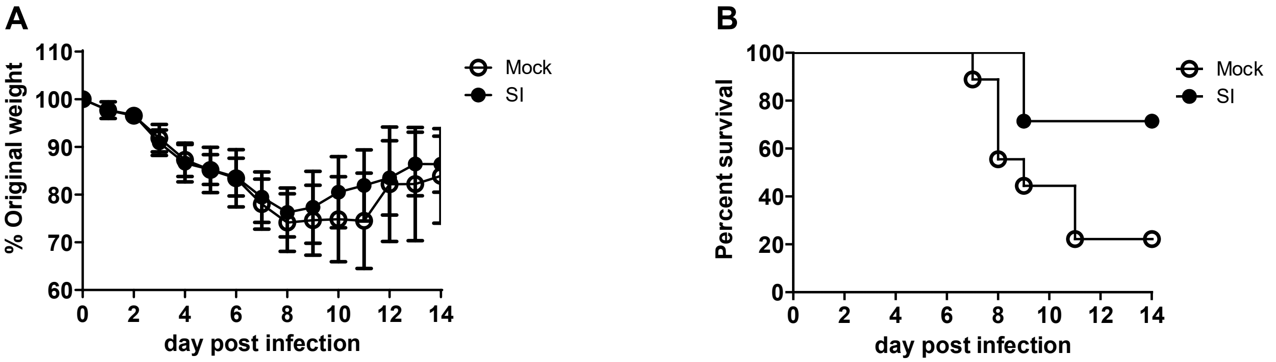

Supplemental Figure 2. 25-month-old mock-infected mice are more susceptible to influenza infection than sequentially infected mice. A. Mock and sequentially infected (SI) mice were monitored for weight loss daily for 14 days. **B.** Mock and sequentially infected (SI) mice were monitored for survival for 14 days after infection. Moribund mice were humanely euthanized. Data are from a single experiment with n = 9 mock infected mice and n = 7 sequentially infected mice.
